# Genetic syndromes screening by facial recognition technology: VGG-16 screening model construction and evaluation

**DOI:** 10.1186/s13023-021-01979-y

**Published:** 2021-08-03

**Authors:** Dian Hong, Ying-Yi Zheng, Ying Xin, Ling Sun, Hang Yang, Min-Yin Lin, Cong Liu, Bo-Ning Li, Zhi-Wei Zhang, Jian Zhuang, Ming-Yang Qian, Shu-Shui Wang

**Affiliations:** 1 Department of Paediatric Cardiology, Guangdong Provincial People’s Hospital, Guangdong Academy of Medical Sciences, Guangdong Cardiovascular Institute, Guangdong Provincial Key Laboratory of South China Structural Heart Disease, Guangzhou, 510000 China; 2 Cardiac Center, Guangdong Women and Children Hospital, Guangzhou, China; 3 Department of Paediatric Cardiology, Shenzhen Children’s Hospital, Shenzhen, China; 4 Department of Cardiac Surgery, Guangdong Provincial People’s Hospital, Guangdong Academy of Medical Sciences, Guangdong Cardiovascular Institute, Guangdong Provincial Key Laboratory of South China Structural Heart Disease, Guangzhou, China

**Keywords:** Facial recognition, Genetic syndrome, Artificial intelligence, Deep learning, Rare diseases

## Abstract

**Background:**

Many genetic syndromes (GSs) have distinct facial dysmorphism, and facial gestalts can be used as a diagnostic tool for recognizing a syndrome. Facial recognition technology has advanced in recent years, and the screening of GSs by facial recognition technology has become feasible. This study constructed an automatic facial recognition model for the identification of children with GSs.

**Results:**

A total of 456 frontal facial photos were collected from 228 children with GSs and 228 healthy children in Guangdong Provincial People's Hospital from Jun 2016 to Jan 2021. Only one frontal facial image was selected for each participant. The VGG-16 network (named after its proposal lab, Visual Geometry Group from Oxford University) was pretrained by transfer learning methods, and a facial recognition model based on the VGG-16 architecture was constructed. The performance of the VGG-16 model was evaluated by five-fold cross-validation. Comparison of VGG-16 model to five physicians were also performed. The VGG-16 model achieved the highest accuracy of 0.8860 ± 0.0211, specificity of 0.9124 ± 0.0308, recall of 0.8597 ± 0.0190, F1-score of 0.8829 ± 0.0215 and an area under the receiver operating characteristic curve of 0.9443 ± 0.0276 (95% confidence interval: 0.9210–0.9620) for GS screening, which was significantly higher than that achieved by human experts.

**Conclusions:**

This study highlighted the feasibility of facial recognition technology for GSs identification. The VGG-16 recognition model can play a prominent role in GSs screening in clinical practice.

## Background

Genetic syndromes (GSs) refer to specific manifestations with multiple clinical features that are caused by genetic abnormalities. Genetic abnormalities can vary from subtle to prominent and from a discrete mutation in a single base on the DNA sequence of a single gene to a gross chromosomal abnormality [1]. Each particular genetic syndrome (GS) presents with characteristic features depending on the developmental aspects affected by the abnormal genes or chromosomes. Although individual cases are rare, GSs collectively affect a significant proportion of the general population, with the majority being children [2, 3]. Children with GSs often suffer repeat admissions, long-term care, and impaired quality of life which may lead to heavy social and family burdens [4].

Timely diagnosis of GSs is crucial for genetic counselling and can improve outcomes. With the development of next-generation sequencing, GS research is becoming extensive, and gene examination is considered the “gold-standard” method for GS diagnosis [5]. However, gene testing is expensive and time-consuming. In clinical practice, gene examination for all patients is unrealistic. Therefore, the main question has become “how can we screen suspected GS patients for further investigation?”

Many GSs have distinct facial dysmorphism, and the recognition of a syndrome from a facial gestalt can be the first step in making a diagnosis [6]. However, due to the variation and complexity in phenotyping, combined with the inexperience of general practitioners, the memorization of different facial gestalts and recognition of rare GSs is a challenging task. Facial recognition technology has been widely applied in several fields, and artificial intelligence has been integrated into routine clinical practice specifically for diagnostic support. With recent advancements in deep convolutional neural networks (CNNs), screening and diagnosis of GSs through facial feature recognition has become possible [7]. In the present study, we developed a facial recognition model based on the VGG-16 architecture (named after its proposal lab, Visual Geometry Group from Oxford University) for identifying GS children from healthy children, and the performance of the model was also evaluated.

## Materials and methods

### Patients and facial photos

A total of 228 children with GSs and 228 healthy children were recruited from Guangdong Provincial People's Hospital from Jun 2016 to Jan 2021. The demographic characteristics of the participants are shown in Table 1.

**Table 1:** Demographic characteristics of children with and without GSs

Characteristic	GSs	Controls	*p*
Number of subjects	228	228	
Age at photograph (months)	36.85 ± 42.33	37.28 ± 40.77	< 0.05
Gender (male/female)	125/103	119/109	< 0.05

*GSs* genetic syndromes

The GS diagnosis was confirmed by karyotyping, array comparative genomic hybridization or next-generation sequencing. The following syndromes were included: Williams-Beuren syndrome (n = 108), Noonan syndrome (n = 52), Down syndrome (n = 14), Marfan syndrome (n = 5), Loeys-Dietz syndrome (n = 4), Alagille syndrome (n = 4), DiGeorge syndrome (n = 3), Acromicric and Geleophysic dysplasia (n = 3), Kabuki syndrome (n = 2), Barth syndrome (n = 2), Cornelia de Lange syndrome (n = 2), Koolen-de Vires syndrome (n = 2). 14q32 duplication syndrome (n = 2). Congenital mental retardation, AD (n = 2). 8p23.1 deletion syndrome (n = 2). 21q22.3 deletion syndrome (n = 2). Helsmoortel-Van der Aa syndrome (n = 1). Mulibrey nanism (n = 1). Congenital fibrosis of extraocular muscles (n = 1). Mandibulofacial dysostosis-microcephaly syndrome (n = 1). Cerebro-oculo-facio-skeletal syndrome (n = 1). Oculo‐facio‐cardio‐dental syndrome (n = 1). Wolf-Hirschhorn syndrome (n = 1). Costello syndrome (n = 1). Cri du Chat syndrome (n = 1). Stickler syndrome (n = 1). Coffin-Siris syndrome (n = 1). Klippel-Feil syndrome (n = 1). Congenital contractural arachnodactyly (n = 1). 16p11.2 duplication syndrome (n = 1). Holt-Oram syndrome (n = 1). X-Linked Oto-palato-digital Spectrum Disorders (n = 1). 16p11.2 microdeletion syndrome (n = 1). Brittle cornea syndrome (n = 1). 18q microdeletion Syndrome (n = 1). In total, there were 35 different genetic syndromes.

**Fig. 1:**
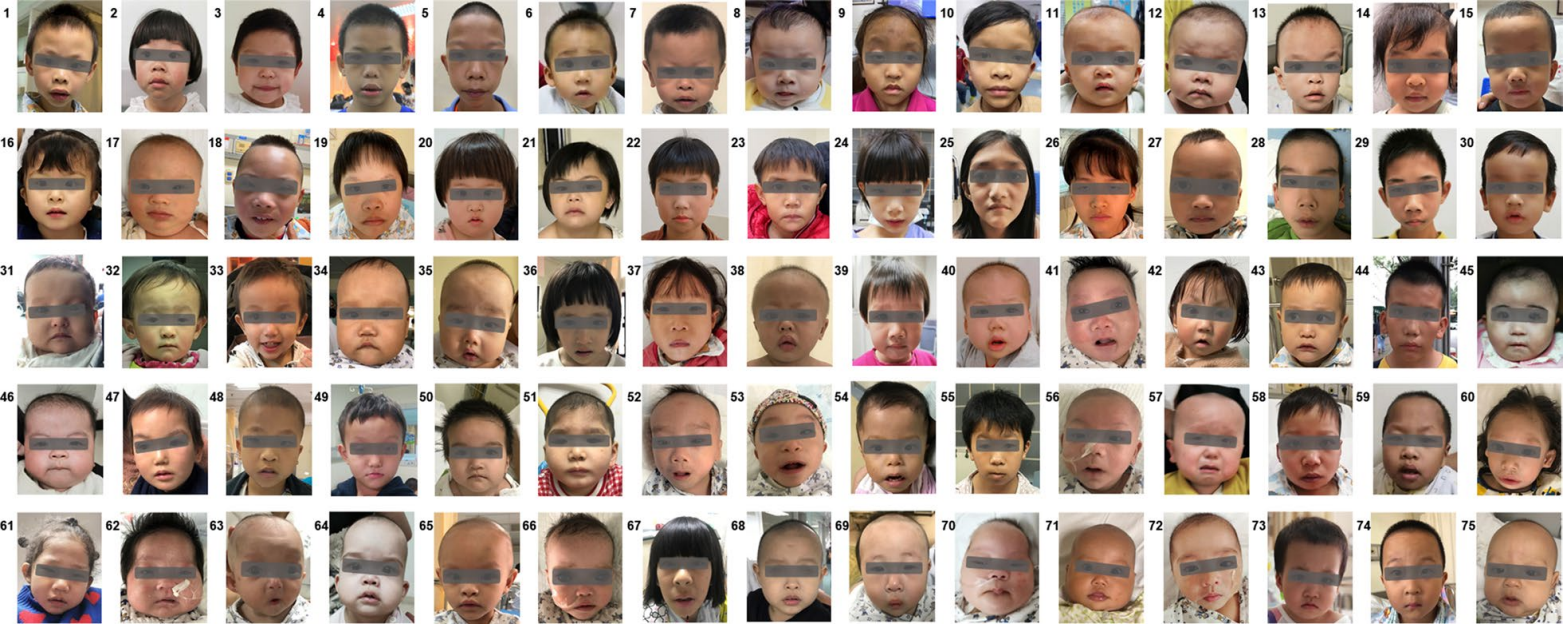
Facial dysmorphism in children with genetic syndromes. Williams-Beuren syndrome (1–7). Noonan syndrome (8–15). Down syndrome (16–21). Marfan syndrome (22–26). Loeys-Dietz syndrome (27–30). Alagille syndrome (31–34). DiGeroge syndrome (35–37). Acromicric and Geleophysic dysplasia (38–40). Kabuki syndrome (41–42). Barth syndrome (43–44). Cornelia de Lange syndrome (45–46). Koolen-de Vires syndrome (47–48). 14q32 duplication syndrome (49–50). Congenital mental retardation, AD (51–52). 8p23.1 deletion syndrome (53–54). 21q22.3 deletion syndrome (55–56). Helsmoortel-Van der Aa syndrome (57). Mulibrey nanism (58). Congenital fibrosis of extraocular muscles (59). Mandibulofacial dysostosis-microcephaly syndrome (60). Cerebro-oculo-facio-skeletal syndrome (61). Oculo‐facio‐cardio‐dental syndrome (62). Wolf-Hirschhorn syndrome (63). Costello syndrome (64). Cri du Chat syndrome (65). Stickler syndrome (66). Coffin-Siris syndrome (67). Klippel-Feil syndrome (68). Congenital contractural arachnodactyly (69). 16p11.2 duplication syndrome (70). Holt-Oram syndrome (71). X-Linked Oto-palato-digital spectrum disorders (72). 16p11.2 microdeletion syndrome (73). Brittle cornea syndrome (74). 18q microdeletion syndrome (75). The grey bar is used to protect privacy

Three to ten frontal facial photos were taken depicting the entire frontal face from hairline to chin, exposing the ears, with opened eyes looking straight ahead. Only one clear frontal facial photo was selected for each participant (avoid those with obvious “open mouth” as much as possible). A total of 456 frontal facial photos were collected from 228 children with GSs and 228 healthy children. Facial images of children with GSs are presented in Fig. 1.

This study was approved by the Research Ethics Committee of Guangdong Provincial Peoples’ Hospital (Project Number: KY2020-033-01). Informed consent was given by all patients or their wardens to analyse.

### Training system

The hardware used for the study was an NVIDIA Tesla P100 GPU (NVIDIA Corporation, California, USA) with 16 GB RAM and 4096 bits. An Ubuntu18.04 operation system (Canonical Ltd, UK) was used. Networks were based on TensorFlow (Google Inc, California, USA).

The study process can be summarized as follows: (1) VGG-16 networks were pretrained through transfer learning methods by VGG-Face CNN descriptors and obtained initializing weights. (2) Face detection from photographs was performed by multitask Convolutional Neural Network (MTCNN), thus achieving five characteristic markers in each photograph. (3) By randomly rotating, cropping or horizontally flipping the detected face, a group of facial images of size 224 × 224 × 3 (RGB) was obtained as the data inputs. (4) A facial recognition model based on the VGG-16 architecture was constructed, and the performance was evaluated by five-fold cross-validation. (5) Gradient-weight class activation mapping (Grad-CAM) was produced to highlight key regions in the facial images, which were processed and recognized by the model. (6) The performance of VGG-16 model was compared to that of five physicians.

### Image pre-processing

MTCNN was used for face detection and alignment. The MTCNN contained an image pyramid and a three-stage cascaded framework: proposal network, refine network and output network, finally generated a facial image (224 × 224 × 3 pixels) with five facial landmark positions (left eye, right eye, nose, left mouth corner, and right mouth corner) for each inputted facial photo. The pixel value of the image was scaled and normalized from 0 to1. The Dataset was augmented by random rotation, cropping and horizontal flipping.

### Transfer learning

We used VGG-16 as our network architecture, and we started transfer learning by initializing the network with pretrained weights from VGG-Face, an open-source face data model supplied by the Oxford Visual Geometry Group (UK). The primary algorithm included softmax for classification training, a triplet loss function for feature extraction training, and the RMSProp optimization method for parameter update.

### Model construction and training

**Fig. 2:**
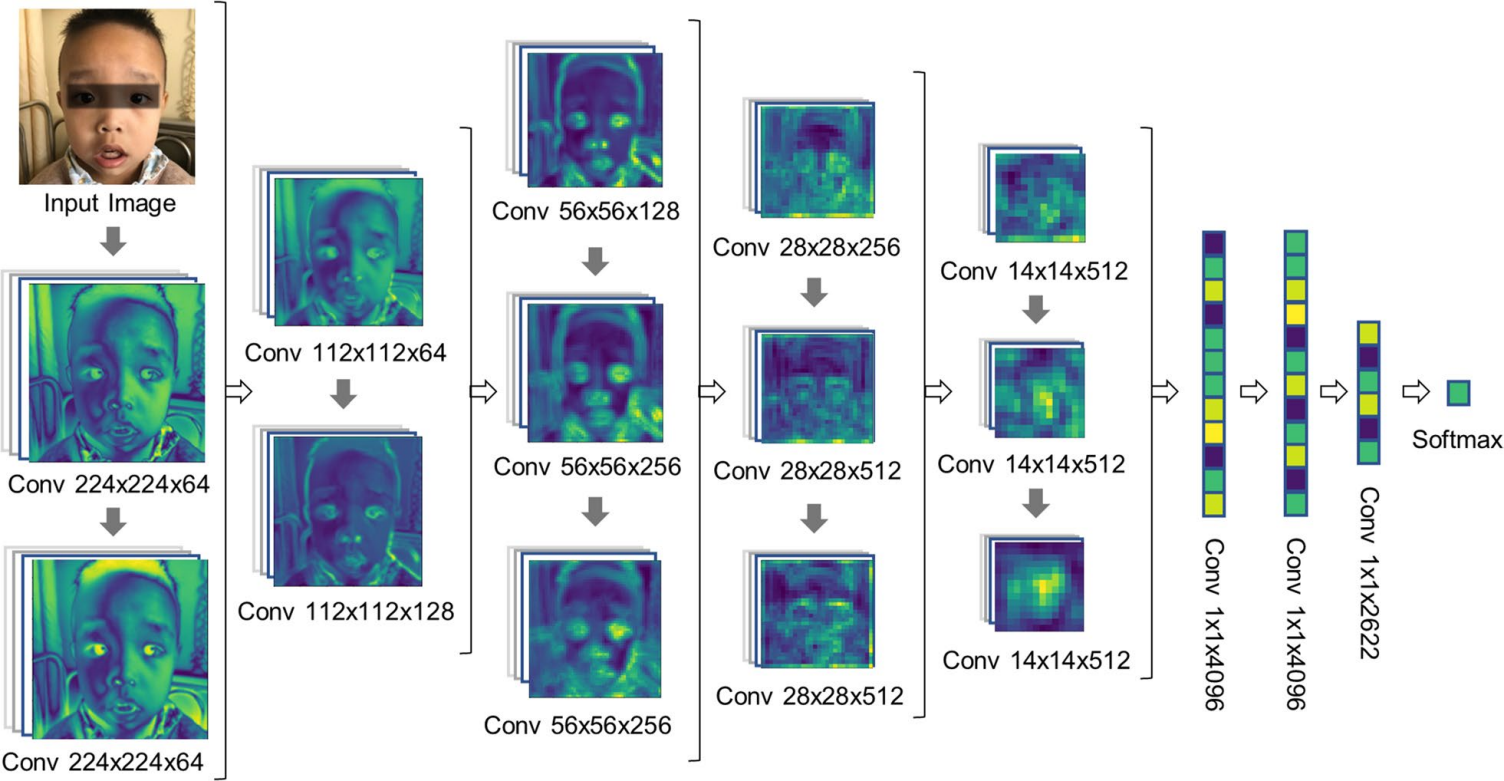
Network architecture of the VGG-16 model. The network consists of 13 convolutional layers followed by three layers with 50% dropout. Five maximum pooling layers are placed after groups of convolution layers. The classification prediction is output by the softmax layer

A facial recognition model based on the VGG-16 architecture was constructed. The VGG-16 architecture comprised 13 convolutional layers, followed by maximum pooling layers, three fully connected layers, and a softmax output. We replaced the fully connected layers with convolutional layers with a 50% dropout. This improvement enhanced the generalization ability, while diminishing the computing capacity and time spent. The convolutional layer convolved the input data and was connected to a rectified linear unit (ReLU) activation function after batch normalization. Following the convolution layer operations, the data were finally outputted via softmax; then, the probability of GS was predicted. A maximum pooling layer placed between two groups of convolution layers to downsample the output data was used to reduce the computational complexity and avoid overfitting. Softmax predicted the probability of input image data being GS-specific faces (Fig. 2).

In the experiment, five-fold cross-validation was adopted. The proportion of the training set, validation set, and test set was 3:1:1. Both the GS and non-GS facial image data were randomly split into five subsets. The GS and non-GS data were distributed equally in each subset.

### Visual explanation

**Fig. 3:**
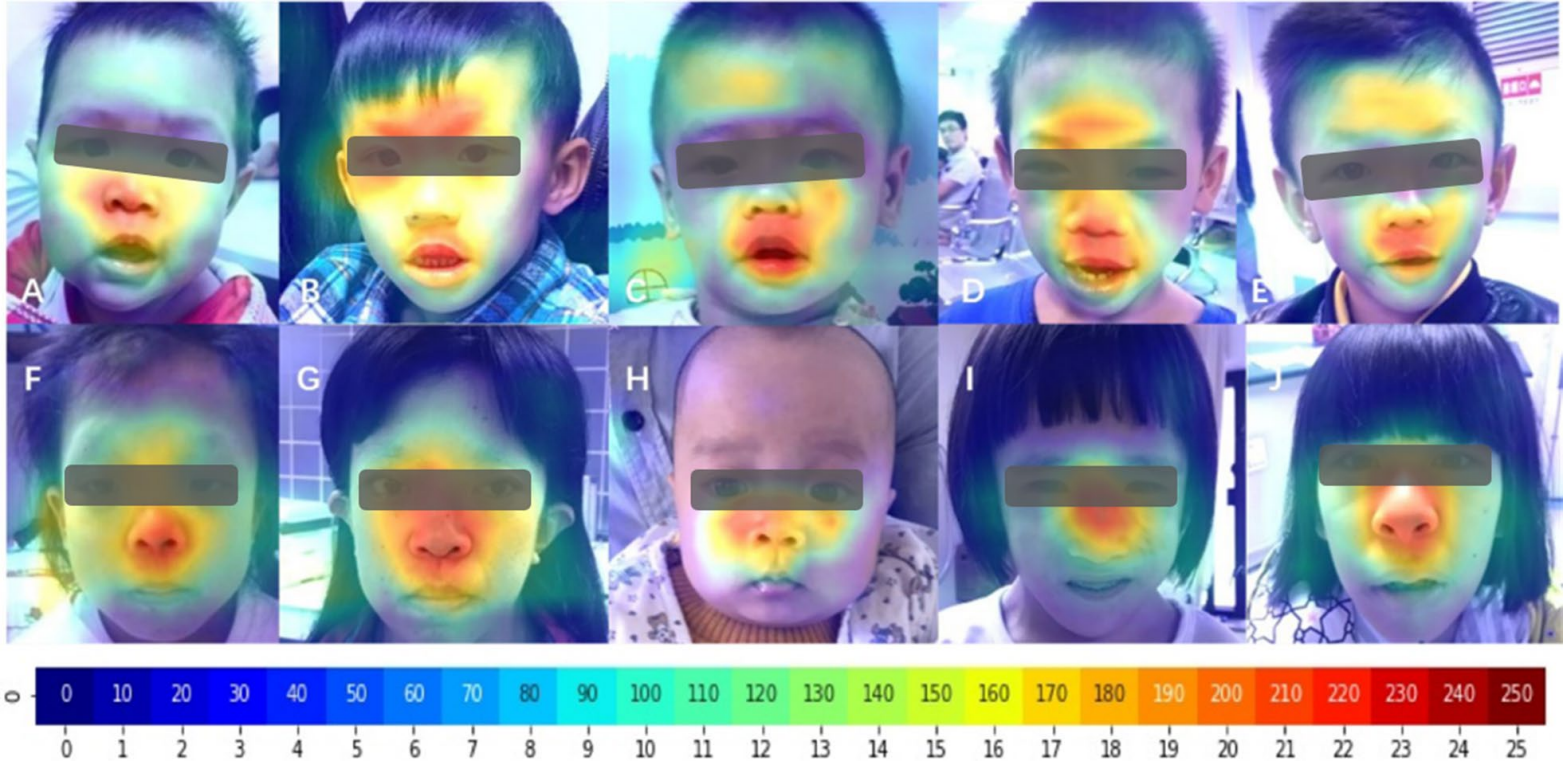
Visualization by Grad-CAM. According to the colour band, the size of the values corresponds to the colour brightness. A higher value corresponds to a brighter expression, thus representing the regions of significant concern. **A**–**E** Williams-Beuren syndrome; **F**–**H** Noonan syndrome; **I** iGeorge syndrome; **J** Coffin-Siris syndrome

To understand the features learned by the VGG-16 model and their locations, we used Grad-CAM to highlight key regions in the facial images that influenced the decision-making by the model (Fig. 3). The code is available at https://github.com/ramprs/grad-cam/, proposed by Selvaraju et al. [8] from the USA.

### Comparison of the model with paediatricians

Three junior paediatricians (those with 3–5 years of experience) and two senior paediatricians (those with more than 15 years of experience) were invited to recognize GS patients based solely on facial photos. One senior paediatrician had received genetics training. The other paediatricians had no experiences with genetic training, but all of them had once managed children with genetic syndromes in daily clinical practice. Each face image was shown for 10 s without exhibiting other clinical data. Based on the photo image from the dataset, the physicians determined whether an individual was suffering from a GS. The classification performance of the VGG-16 model was compared to these five paediatricians.

### Evaluation metrics

Identification results were noted as TP (true positive), FP (false positive), TN (true negative), and FN (false negative). The classification performance of the proposed VGG-16 model was quantified by accuracy, recall, specificity, precision, F1-score, and area under the receiver operating characteristic curve (AUC). The identification performance of the paediatricians was quantified by accuracy, sensitivity (the same as recall), specificity, precision, and F1-scorec. These measures were calculated as follows:

$$Accuracy \, = \frac{TP + TN}{{TP + FP + TN + FN}}$$

$$Specificity \, = \frac{TN}{{TN + FP}} = 1 - \frac{FP}{{TN + FP}}$$

$$Recall \, = \frac{TP}{{TP + FN}}\; ( = \,sensitivity)$$

$$Precision \, = \frac{TP}{{TP + FP}}$$

$$F1 - score \, = \frac{2}{{\frac{1}{precision} + \frac{1}{recall}}}$$

### Statistical analysis

Model performance measurements were reported as the mean ± standard deviation of five testing results obtained from the cross-validation. Receiver operating characteristic curve (ROC) with 95% confidence interval (CI) of VGG-16 model was calculated and plotted by using package pROC 1.17.0.1 in R 3.6.1 with 200 iterations of bootstrapping. To compare the classification performance of the VGG-16 model and physicians, the sensitivity/specificity point of each physician was plotted on the ROC space of the VGG model. When the sensitivity/specificity point of physician lies outside of the 95% CI space of the ROC curve of VGG model, the classification performance of VGG model and physician are defined as statistically difference [9]. Pearson’s chi-squared-test was applied to compare the gender proportions, and an independent-sample t-test was used to compare the age at photograph between the groups. *P*-values < 0.05 were considered statistically significant.

## Results

### Model performance

**Fig. 4:**
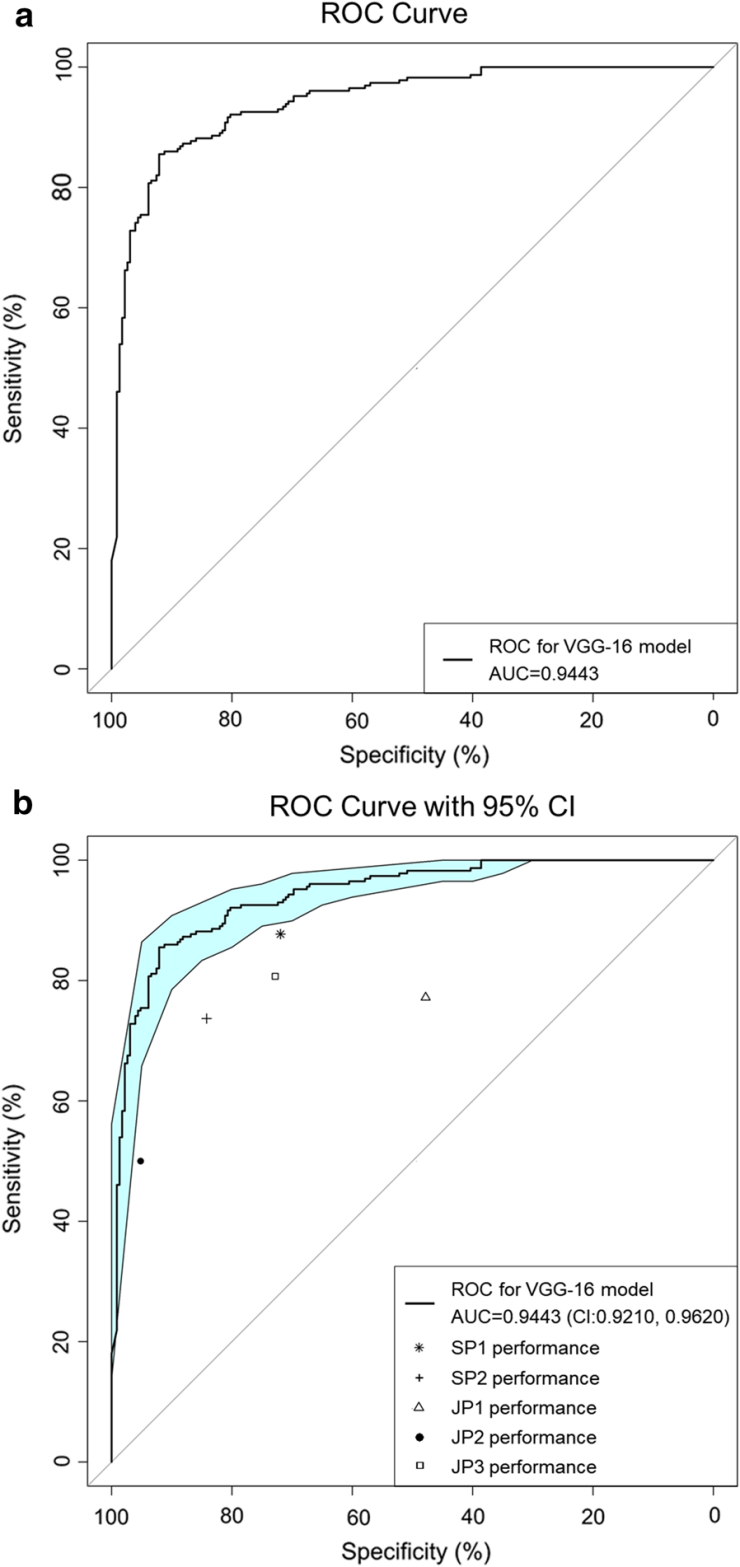
Receiver operating characteristic (ROC) curve of the VGG-16 model, together with performance of human experts plotted on the same ROC space. **a** ROC curve and area under the ROC curve value of the VGG-16 model. **b** The sensitivity/specificity point of each physician was outside of the 95% confidence interval space of the ROC curve of VGG model. *AUC* area under the ROC curve, *CI* confidence interval, *SP* senior paediatrician, *JP* junior paediatrician

The VGG-16 model achieved an accuracy of 0.8860 ± 0.0211 and an AUC value of 0.9443 ± 0.0276 (Fig. 4). Other model performance measurements are given in Table 2.

**Table 2:** Identification performance of paediatricians and VGG-16 model

	Accuracy	Sensitivity (Recall)	Specificity	Precision	F1-score
SP 1	0.7983	0.8772	0.7193	0.7576	0.8130
SP 2	0.7895	0.7368	0.8421	0.8235	0.7778
JP 1	0.7259	0.5000	0.9518	0.9120	0.6459
JP 2	0.6250	0.7719	0.4781	0.5966	0.6730
JP 3	0.7675	0.8070	0.7281	0.7480	0.7764
Model	0.8860 ± 0.0211	0.8597 ± 0.0190	0.9124 ± 0.0308	0.9079 ± 0.0315	0.8829 ± 0.0215

*JP* junior paediatrician, *SP* senior paediatrician

### Visual explanations by feature maps

Weighted feature maps were computed by the ReLU activation function, reserving the class features and abandoning the unrelated features; then, the values were normalized into the range 0–255. From the colour band, the size of the values corresponded to the colour brightness. In most cases, the expression was brighter for higher values, and it represented relatively more significant regions on the face (Fig. 3). Class activation maps matched the dysmorphic facial features well in 217 GS images. In the other 11 GS photos, the class-discriminative regions were focused not only on the facial regions, but also on the hair or clothes.

### Comparison with human experts

The performance results of the five paediatricians are shown in Table 2. One of the senior paediatrician, who had genetics training experience, achieved the best accuracy (0.7983) and sensitivity (0.8772). The sensitivity/specificity point of each physician was outside of the 95% CI space of the ROC curve of VGG-16 model, indicating that the identification performance of each participating paediatrician was inferior to that of the VGG-16 model (Fig. 4).

## Discussion

GSs often present with characteristic phenotypes that include dysmorphic features and characteristic facial gestalts. These craniofacial alterations can provide clinicians with important diagnostic clues. For instance, Down syndrome has a disease-specific facial profile that can be recognized easily. There are approximately 7000 genetic syndromes, the vast majority of which are rare diseases, and the characteristic craniofacial features are often unfamiliar to general physicians and paediatricians. However, with technical advancement in computing, GS facial recognition is becoming easily available. Loos et al. [10] first reported that GSs can be identified by using facial resemblance and a traditional machine learning method, with an accuracy of 83%. With improvements in data storage and computational power, deep CNN has become the most important facial recognition method.

In 2014, Face2Gene (http://www.face2gene.com/, FDNA Inc., Boston, USA), based on the DeepGestalt framework (one of the deep CNN algorithms), was introduced for GS facial recognition [11]. When a facial photo is uploaded, Face2Gene produces a ranked list of 30 types of possible GSs. The performance of Face2Gene is evaluated using “top-10 accuracy”, which is the likelihood that one of the 10 syndromes with the highest probabilities suggested by Face2Gene is the actual syndrome. Studies showed that Face2Gene can help discriminate between different types of GSs [12, 13]. Hsieh et al. [14] introduced an approach that used portrait photographs for the interpretation of clinical exome data. This study indicated that image analysis by DeepGestalt could quantify the phenotypic similarity to advance the performance of bioinformatics pipelines for exome analysis. However, each uploaded facial image is defaulted as “abnormal” by Face2Gene. Even if a photograph of a healthy child is input into Face2Gene, a list of 30-type candidate GSs is produced, implying that the software lacks a screening function. Hence, developing a facial recognition model for screening GSs is necessary. In 2020, Pantel et al. [15] analysed a total of 646 images of 323 patients with 17 different genetic syndromes and matched individuals without a genetic syndrome. A face recognition model, which is driven by support vector machine running on the top of DeepGestalt framework, was introduced in this study. This novel approach could fairly separate images of individuals with and without a genetic syndrome.

VGG-net, proposed by the Visual Geometry Group (VGG) Lab of Oxford University, is a popular CNN architecture. VGG-16 is characterized by its simplicity in using only 3 × 3 convolutional layers stacked on top of each other in increasing depth. The increased depth and smaller kernel can diminish the network parameters, thus promoting the fitting capacity and wide clinical application. This network has been widely applied in computer vision fields. Recently, the medical applications of VGG-16 have been reported. Related works cover areas on the identification of tumour properties, disease staging on medical image data, retinal fundus image interpretation, etc. [16–20]. We constructed a facial recognition model using the VGG-16 architecture for GS screening. The model proposed in this study achieved high performance, with an accuracy of 0.8860 ± 0.0211 and an AUC of 0.9443 ± 0.0276. The proposed VGG-16 screening model has excellent performance in discriminating GS children with non-GSs, and outperformed all the participating paediatricians with statistical significance. Visual explanations via Grad-CAM can provide insights into dysmorphic facial characteristics. However, limited dataset and indiscernible image details may influence the localization ability of Grad-CAM.

The quality of a CNN model is dependent on the size of the dataset. Due to the low incidence of GSs, the number of dysmorphic facial photographs has often been limited, which risks the deep CNN model overfitting in cases of small datasets. The transfer learning method can solve this problem. Transfer learning is the reuse of a pretrained model on a new problem. It enables researchers to benefit from the knowledge gained from a previously used model for a similar task, analogous to humans’ capacity to use previously acquired knowledge to solve a similar problem [21]. The transfer learning technique has often been used with small sample studies. Zhen et al. [22] reported research on predicting rectum toxicity in patients receiving radiotherapy for cervical cancer. Transfer learning from substantial natural images has solved the problem of limited data. In the current study, the VGG-16 networks had pretrained weights from the large-scale face dataset “VGG-Face” for learning low-level visual features from the general population. Therefore, the model parameters were fine-tuned by using our facial image dataset and gained knowledge of high-level visual features in GS facial manifestations.

In this study, we gathered 228 cases with 35 different GSs. There are many typical but rare dysmorphic facial images in this facial photograph dataset. These craniofacial alterations can provide clinicians with important diagnostic clues, and an automatic facial recognition model for GS screening can be constructed using these facial images. However, there were several limitations in the study. (1) A good diagnostic model is often based on a sufficiently large and general dataset. As most GSs are rare diseases, the facial photos training set in this study was limited, and it will be beneficial to collect more GS cases. (2) All participants were from East Asia. There were no Caucasian, African, or other ethnic cases enrolled in the study. Facial dysmorphic features may be influenced by ethnic backgrounds. (3) Enrolled children were mainly composed of toddlers or preschool children. Therefore, the proposed model in the current study may not be appropriate for infants, neonates, or adults.

## Conclusions

This study highlighted the feasibility of facial recognition technology for GSs identification. The VGG-16 recognition model can play a prominent role in GSs screening in clinical practice.

## Data Availability

The datasets supporting the conclusions in this article are primarily included within the article and available from the corresponding authors upon reasonable request.

## Abbreviations

GSs: Genetic syndromes
GS: Genetic syndrome
CNN: Convolutional neural networks
Grad-CAM: Gradient-weight class activation mapping
ReLU: Rectified linear unit
ROC: Receiver operating characteristic
AUC: Area under the ROC curve
VGG: Visual Geometry Group
